# Complementary Feeding Strategies to Facilitate Acceptance of Fruits and Vegetables: A Narrative Review of the Literature

**DOI:** 10.3390/ijerph13111160

**Published:** 2016-11-19

**Authors:** Sophie Nicklaus

**Affiliations:** Centre des Sciences du Goût et de l’Alimentation, CNRS, INRA, Univ. Bourgogne Franche-Comté, 17 Rue Sully, Dijon F-21000, France; sophie.nicklaus@inra.fr

**Keywords:** nutrition, complementary feeding, fruit, vegetables, eating behavior, repeated exposure, breastfeeding, feeding practices

## Abstract

Complementary feeding (CF), which should begin after exclusive breastfeeding for six months, according to the World Health Organization (WHO), or after four months and before six months according to the European Society for Pediatric Gastroenterology Hepatology and Nutrition (ESPGHAN), is a period when the infant implicitly learns what, when, how, and how much to eat. At the onset of CF, the brain and the gut are still developing and maturing, and food experiences contribute to shaping brain connections involved in food hedonics and in the control of food intake. These learning processes are likely to have a long-term impact. Children’s consumption of fruit and vegetables (FV) is below recommendations in many countries. Thus, it is crucial to establish preferences for FV early, when infants are learning to eat. The development of food preferences mainly starts when infants discover their first solid foods. This narrative review summarizes the factors that influence FV acceptance at the start of the CF period: previous milk feeding experience; timing of onset of CF; repeated exposures to the food; variety of foods offered as of the start of the CF period; quality and sensory properties of the complementary foods; quality of the meal time context; and parental responsive feeding.

## 1. Introduction

Complementary feeding (CF), which should begin after exclusive breastfeeding for six months, according to the World Health Organization (WHO) [1], or after four and before six months according to the European Society for Pediatric Gastroenterology Hepatology and Nutrition (ESPGHAN) [2], is a period when the infant learns implicitly what, when and how to eat and how much of a given food to eat. At the onset of CF, the brain and the gut are still developing and maturing, and food experiences contribute to shaping brain connections involved in food hedonics and in the control of food intake. These learning processes are likely to have a long-term impact on eating behavior [3,4,5,6]. By the end of the second year, food neophobia, i.e., the refusals of new foods, develops [7,8,9]. Thus, it is important to understand the determining factors of the acceptance of the first foods other than milk, since they form the basis of the child’s future food repertoire, and their acceptance will be more difficult to promote later in childhood. 

Despite recommendations throughout Europe to increase children’s consumption of fruit and vegetables (FV), they remain below recommendations in many countries [10]. For instance, in France, the National Program on Nutrition and Health recommends consuming five 80-gram portions of fruit and vegetables per day [11], but the consumption observed in three- to 10-year-old children in 2007 only reached 74 g for fruit and 67 g for vegetables [12]; FV consumption below recommendations was also observed in Portugal, the UK, and Greece [13]. Thus, it is crucial to establish preferences for FV early, when infants are learning to eat. We will summarize the factors that influence FV acceptance at the start of the CF period: previous milk feeding experience; timing of onset of CF; repeated exposures to the food; variety of foods offered as of the start of the CF period; quality and sensory properties of the complementary foods; quality of the meal time context; and parental responsive feeding.

## 2. Role of Previous Mode of Milk Feeding

The type of milk feeding mode chosen by the mother (breast vs. bottle) may influence the acceptance of foods at later stages in a variety of ways: it may modify the development of food and flavor acceptance, of oral feeding skills, and of infant control of energy intake, as will be developed here.

First one may consider that the flavor exposure received by the infant differs between breastfeeding and bottle feeding. Breast milk flavors may vary from one feeding episode to another [14,15]. Formula flavors may also differ according to their types, whether they are regular, antiallergic (with hydrolyzed protein), antidiarrheic, fermented, etc. [16]. The milk feeding mode may be associated with a differential food acceptance at the beginning of CF: breastfeeding is associated with a higher acceptance of a new food during the first days of the CF period [17,18,19], or within one month after the beginning of CF [20]. However, this association is not observed when acceptance is averaged over a two-month period [21]. Moreover, after two weeks of exposure to a flavored food, breastfed and bottle-fed infants do not differ in their acceptance of this food compared to an unflavored version [20]. The positive impact of breastfeeding on further food acceptance may be mediated by the variety of flavor exposure in the milk context [17,20]. This impact may be limited to the very beginning of CF. The infant’s taste experience in the milk feeding context may modify further food preferences. The longer the breastfeeding duration, the higher the acceptance of a umami-tasting solution at the age of six months [22]. Exposure to hydrolyzed-protein formulas is associated with a different taste preference pattern later, up to the age of five years [23,24].

Epidemiological studies also revealed that breastfeeding duration is positively associated with food variety later: it is associated with a variety of free food choices by two- to three-year-old children [25], with healthy eating habits at two years [26], with food variety at two years [27], with fruit consumption at six to eight years [28] and with a healthy eating pattern at two to eight years [29]. A longer breastfeeding duration was consistently related to higher FV intake in two- to four-year-old children, as shown with data from four European birth cohorts [13]. Exclusive breastfeeding for at least three months is associated with a higher consumption of vegetables at four years [30]. In particular, it was shown that the maternal dietary variety (especially for the fruit and vegetable group) during early childhood, but not during pregnancy, predicted the acceptance of fruit and vegetables in children [31]. Thus, in addition to breastfeeding, the FV consumption by the mother may be important for enhancing FV acceptance in children.

Furthermore, the positive effect of breastfeeding on acceptance of complementary foods may also be related to its impact on feeding skills. Children who were breastfed for at least 12 months had better masticatory functions at three to five years [32]. Sucking from the breast induces a stronger aspiration and intra-buccal depression, requiring stronger activity of the lips and propulsion/retro-propulsion movements of the mandible compared to sucking from a bottle [33]. A reduction in masseter activity was indeed observed during bottle feeding compared to breastfeeding [34,35,36,37]; ultimately this different organization may affect craniofacial growth and oral functions [32,38]. The impact of breastfeeding on the duration and number of chewing cycles required to eat solid, viscous and puréed food in infants aged between six and 24 months has not been systematically examined [39]. However, a review of the effects of the mode of milk feeding on oral motor development concluded that starting with breastfeeding may promote a proper oral motor development [40], but this would require further experimental support, in particular to examine if this may specifically influence the acceptance of fruit and vegetables at the initiation of CF. 

Third, breastfeeding is also associated with a different influence on food intake control abilities from bottle feeding. Contrary to bottle feeding, breastfeeding does not make it possible for mothers to see and control the ingested amount of milk [41]. Consequently, it was hypothesized that bottle feeding could be one of the early feeding practices likely to alter innate food intake control abilities [42,43,44,45]. Specifically, bottle feeding may influence these abilities in the short term [43] and at the age of six years [44]. This may also contribute to a differential acceptance of food between breastfed and bottle-fed infants at the beginning of CF [17,18,19], but the involved mechanisms should be explored more specifically. 

## 3. Role of the Timing of Complementary Feeding

Because of physiological and/or psychological development, one or more specific time windows may lead to an easier acceptance of complementary foods, or would lead to more sustainable preferences [46,47]. However, the consequences of the timing of onset of CF on eating behavior and acceptance are not well documented [48], since most investigations have focused on the nutritional consequences of the cessation of breastfeeding.

Concerning solid foods, the acceptance of cereals with an unknown flavor compared to plain cereals was higher in infants aged 16–17 weeks than in infants aged 18–25 weeks [49]. In an observational study conducted in France with the OPALINE birth cohort (Observatory of food preferences in infants and children, *n* = 203), it was showed that infants from mothers who introduced vegetables earlier in the CF process (i.e., between four and five months of age) accepted more of the vegetables they were offered [21]. This effect was observed only for the vegetable category, which was in this study among the first food categories offered to the infants, but not for the fruit category, another food category introduced early in the CF process. Thus, it is difficult to fully conclude the effect of the timing of CF on the acceptance of complementary foods.

The consequences of an early introduction to FV have been analyzed in different ways. One clinical study reported a positive relationship between early consumption of fruit and measured fruit intake at the age of 18 months [50]. An observational study based on parental reporting revealed that in the UK, early introduction to FV (with no indication of specific age) was associated with a higher consumption of FV, respectively, at two to five years of age [51]; however, for vegetables the effect of early introduction was no longer significant after adjustment for parental vegetable intake or parental neophobia. An observational study conducted in the USA showed that the association between the early introduction to fruit and the later consumption of fruit was stronger than between the early introduction to vegetables and the later consumption of vegetables [28]. One might conclude that it is not necessary to introduce vegetables early in the infant’s diet. However, vegetables are much less energy-dense than fruit and other foods [52], which does not favor their consumption in young children [53]. Therefore, it may be advised to introduce vegetables early in the CF process in order to promote their acceptance. Clearly, intervention trials are needed to address this issue more specifically, and to assess the long-term effect of timing of FV introduction on future consumption.

A recent experimental study explored whether there may be a benefit of introducing vegetables before fruit, compared to the introduction to fruit before vegetables, by randomizing participants into two groups with initiation of fruits before vegetables or vegetables before fruits [54]. It showed that repeated exposure to fruit had no effect on the vegetable intake, and vice versa. However, because vegetable intake was lower than fruit intake from the start, and because reported daily intake of vegetables at the age of 12 months was 38% higher in infants first fed with vegetables than in infants first fed with fruit, the authors suggest that CF should start with vegetables in order to promote vegetable acceptance in infants. However, the reported daily intake of vegetables at the age of 23 months was similar in both groups [55].

Altogether, it may be that the introduction of vegetables early in the CF process may present an advantage in terms of later acceptance of these foods, but this also raises the question of “how early” should such foods be introduced to the infant’s diet. Exposure to solid foods before the fourth month of age may increase the risk of allergic diseases [2,48,56]. However, there is no reason to delay the introduction of these foods after the age of four months [56]; therefore, fruit and vegetable introduction should start early after the age recommended for the initiation of CF, that is, not before 17 weeks and not after 26 weeks of age [2,56].

## 4. Role of Repeated Exposures

The repetition of exposure to a food is one of the primary determinants of its acceptance. For many types of stimulations (auditory, visual, etc.), providing repeated exposure to them increases their familiarity, which is associated with a shift in the hedonic judgment toward them [57]. This was also shown to be true concerning foods, as an increase in familiarity with a food reduces neophobic reactions and increases hedonic evaluation in children [58] and even in adults [59]. 

In infants, several studies have shown that a food is consumed more and is judged as more liked by the infant after several offers: an increase in acceptance of a new green vegetable was observed after 10 exposures to this food [19]. An increase in intake of a new fruit or vegetable was also shown after eight exposures [60]. The effect of repeated exposure is potent enough to increase the acceptance of foods which had been previously identified by the mother as being refused by her infant during the beginning of the CF process, which were most often green vegetables, but also pumpkin [61]. However, despite the efficacy of this mechanism, foods are most often only presented a limited number of times (often less than five times) before the parent(s) decide that the infant dislikes this food [62,63].

In infants at the start of CF, repeated exposure was as effective as associating a new vegetable with a liked flavor (the sweet taste) to increase its intake, whereas associating it with a higher energy content (addition of oil) did not increase its intake, probably through learned satiation [64]. This suggests that the repeated exposure mechanism is as effective as and simpler to implement than flavor-flavor learning and more effective than flavor-nutrient learning for increasing vegetable acceptance at CF. 

## 5. Role of the Variety of Foods Offered

As described previously, repeated offerings of a given food may enhance its acceptance. Moreover, repeated offerings of a variety of foods may also promote the acceptance of an unknown food. Infants aged approximately six months were shown to better accept carrot, a new food, either if they had been repeatedly exposed to carrot (repeated exposure effect) or to a variety of foods differing from one day to the next, but not if they had been repeatedly exposed to potato [65]. Moreover, infants exposed to a variety of foods better accepted chicken than children exposed to potato or to carrot. Other studies showed that the effect of “exposure to food variety” enhanced the acceptance of green beans (a less liked food than carrot), only if the exposure to variety was higher than the variety necessary to increase the acceptance of carrot, i.e., if pairs of different foods were presented over several days rather than if a different food was presented on each day [66]. So variety might be applied from one day to the next [18] or within a meal [66]. Thus, introducing more than one food per eating occasion might be a way to enhance exposure to flavor variety, and therefore acceptance of new foods. Moreover, the benefit of introducing a variety of vegetables at the beginning of complementary feeding maintains at least up to the age of six years, as was recently shown [67]. Furthermore, the efficiency of the exposure to variety effect may depend on the age of the infant at the beginning of CF [68]: among infants introduced to CF after 5.5 months, the acceptance of a new vegetable was higher in infants previously exposed to a variety of foods than in infants previously exposed to a single food. Altogether, these findings suggest that exposure to variety is a robust mechanism favoring the acceptance of new foods. 

A randomized controlled trial was conducted in UK, Greece and Portugal to investigate whether exposure to a wide variety of vegetables early in the CF process would prevent the observed decline in liking and intake of vegetables at a later age [69]. Parents were randomized to receive shortly before the start of CF either (i) guidance on introducing a variety of culturally appropriate vegetables as first complementary foods or (ii) usual care. Infants in the intervention group showed increased consumption and liking of an unfamiliar vegetable in the short term, but only in the countries where single vegetables were not among the common first foods offered to infants (i.e., in UK and Greece). The effect of the intervention on consumption and liking of an unfamiliar vegetable was not maintained at the six- or nine-month follow-ups. 

As described above, there may be a benefit of exposure to a variety of foods early after the onset of CF in terms of acceptance of unfamiliar foods; however, in the case of food allergy development, the introduction of a variety of foods may make the identification of the food responsible for the allergy more difficult [56].

## 6. Impact of the Sensory Properties of Foods on Their Acceptance

The sensory properties of FV are important determinants of their initial acceptance by infants, in particular texture, taste and aromatic properties. Due to the limited oral skills of the infant, texture is one of the properties that requires the most adaptation to enable the infant to ‘process’ and swallow the food [70]. A significant proportion of infants (23%) have difficulties with foods containing pieces [71]. However, these difficulties should not be interpreted by parents as a reason to delay the introduction to solid foods. Delayed introduction is indeed associated with further problems of texture acceptance: offering new and varied textures before or at nine months is associated with less food refusals and a better acceptance of complementary foods later on than offering varied textures after 10 months [72]. The best predictor of acceptance of chopped carrots in 12-month-old infants is the infant’s previous experience with carrots presented under a variety of textures [73]. Introduction to lumpy foods before the age of six months was associated with fewer food refusals at seven years, and a higher FV consumption [74]. 

Taste may also impact the acceptance of new foods at the beginning of CF. By analyzing foods offered to infants aged five to seven months, it was shown that reactions to new vegetables were more positive if the vegetable was salted or contained a salty ingredient [75]. However, this observation should not encourage parents to use salt or salty ingredients, because sodium is not recommended for infants [2,76]. 

The contribution of flavor as a whole to the initial acceptance of vegetables can be interpreted by comparing the effect of exposure to a variety of vegetables on the acceptance of carrot [65] or of green beans [66]. Acceptance of green beans appears more difficult to promote than that of carrot, no doubt in part due to the difference in the tastes of the two vegetables, with one being sweeter than the other. In some repeated exposure studies, different vegetables were used: the acceptance of green beans is easier to enhance through repeated exposure than the acceptance of artichoke [54].

Interestingly, one study evaluated the effect of combined strategies on the acceptance of vegetables, by intervening on the quality of flavor exposure, through a progressive introduction of flavor variety in the infant diet, first in milk, then in cereals [77]. Infants who received a variety of flavors in milk, then in rice cereal, liked and ate the target vegetable purees more than infants with regular care immediately after the intervention; however, the effect was not sustained at the six-month and 18-month follow-ups, providing evidence of a short-term effect of flavor exposure on vegetable acceptance.

## 7. Impact of Quality of the Meal Time Context and of Parental Responsive Feeding

Acceptance of FV may be learned in interactions with contextual signals from the eating environment. Infants are not able to self-feed, nor to make appropriate food choices by themselves; thus all their meals over several years take place in a social context, where at least one caregiver is present. This external control of the meals by the parents determines the types of foods children are exposed to: in this “passive” way, feeding practices shape early learning through the mechanisms previously described. 

However, feeding FV may be associated with parents’ emotional signals as well as verbal instructions that in turn are likely to modify more actively the child’s eating behavior, and possibly his enjoyment of the food(s) consumed [78]. Social interactions may impact children’s behavior quite early: an experimental study conducted in the USA showed that the volume of formula ingested by infants aged seven to 14 weeks was higher in situations with social interactions [79]. The quality of social interactions is also likely to be associated with a differential acceptance of foods. For instance, parenting style is as likely to impact the child’s enjoyment of eating as the food itself: a survey conducted in France showed that parents who were the most permissive in terms of child feeding had young children with high levels of pickiness, neophobia, and with lower appetite and enjoyment of foods [80]. In such association studies, the causality is difficult to infer. Previous reviews have synthesized the role of parenting style [81] or other feeding practices on the development of eating behavior [82], focusing on the role of external reward given by parents on food acceptance in children according to the type of rewards [83], or on the association between parenting style and FV consumption [84]. 

The importance of these interactions in the feeding situation was long recognized by Satter in the “Division of Responsibility” approach, which states that “parents manage the what, when, and where of feeding and allow children to determine the how much and whether of eating” [78,85]. This social aspect of eating is further taken into account in the concept of responsive parenting, and in particular responsive feeding, which reflects reciprocity between the child and the caregiver, and is now viewed as a promising way to promote healthy eating habits [86]. Intervention trials recently showed that providing parents with an educational approach to promote responsive parenting is associated with slower weight gain in the first year [87]. Thus, responsive parenting practices may be a promising way to promote healthy caring practices. More intervention trials in this area should help to understand whether providing information about responsive parenting may have an effect on feeding practices, on children’s preference for healthy foods, and ultimately on children’s health status in a variety of cultural contexts. 

## 8. Conclusions

This narrative review aimed to describe how the acceptance of fruit and vegetables at the beginning of complementary feeding could be enhanced through different feeding strategies. Some studies highlighted that there may be strong inter-individual differences in reactions to these different strategies. It was shown that infants with specific eating temperaments may be resistant to learning to accept new food: for instance, higher fussiness in infants and toddlers predicted a lower increase in vegetable intake in a learning trial [88]. Moreover, infants with a different taste or olfactory sensitivity may also react differently to vegetables: some infants were more prone to accepting foods (in particular vegetables) with a sweet, sour or savory taste [75]; some infants who were highly reactive to food odors may show higher dislike for these foods [89,90]. Nevertheless, several strategies appear promising in promoting toddlers’ FV consumption. They can be summarized as follows: breastfeeding, especially if the mothers eat a variety of FV while breastfeeding; introducing FV early in the CF process; repeating the presentation of a given FV several times, even if it appears to be initially disliked; introducing a variety of FV; offering FV in an appropriate way to make their sensory characteristics appealing to infants, and to slightly reinforce their energy density; applying responsive feeding practices. However, for some strategies, more randomized-control trials are needed to examine their effectiveness to promote a higher acceptance of fruit and vegetables during the complementary feeding period and during further developmental stages. 
